# Total Knee Replacement and the Effect of Technology on Cocreation for Improved Outcomes and Delivery: Qualitative Multi-Stakeholder Study

**DOI:** 10.2196/jmir.7541

**Published:** 2018-03-20

**Authors:** Yasmin van Kasteren, Jill Freyne, M Sazzad Hussain

**Affiliations:** ^1^ Adaptive Social and Economic Systems Land and Water Commonwealth Scientific and Industrial Research Organization Dutton Park, QLD Australia; ^2^ Australian e-Health Research Centre Health and Biosecurity Commonwealth Scientific and Industrial Research Organization Epping, NSW Australia

**Keywords:** arthroplasty, replacement, osteoarthritis, patient participation, consumer health informatics, technology, telemedicine, rehabilitation, self-care, exercise therapy, human computer interaction, wearables

## Abstract

**Background:**

The growth in patient-centered care delivery combined with the rising costs of health care have perhaps not unsurprisingly been matched by a proliferation of patient-centered technology. This paper takes a multistakeholder approach to explore how digital technology can support the cocreation of value between patients and their care teams in the delivery of total knee replacement (TKR) surgery, an increasingly common procedure to return mobility and relieve pain for people suffering from osteoarthritis.

**Objective:**

The aim of this study was to investigate communications and interactions between patients and care teams in the delivery of TKR to identify opportunities for digital technology to add value to TKR health care service by enhancing the cocreation of value.

**Methods:**

A multistakeholder qualitative study of user needs was conducted with Australian stakeholders (N=34): surgeons (n=12), physiotherapists (n=3), patients (n=11), and general practitioners (n=8). Data from focus groups and interviews were recorded, transcribed, and analyzed using thematic analysis.

**Results:**

Encounters between patients and their care teams are information-rich but time-poor. Results showed seven different stages of the TKR journey that starts with referral to a surgeon and ends with a postoperative review at 12 months. Each stage of the journey has different information and communication challenges that can be enhanced by digital technology. Opportunities for digital technology include improved waiting list management, supporting and reinforcing patient retention and recall of information, motivating and supporting rehabilitation, improving patient preparation for hospital stay, and reducing risks and anxiety associated with postoperative wound care.

**Conclusions:**

Digital technology can add value to patients’ care team communications by enhancing information flow, assisting patient recall and retention of information, improving accessibility and portability of information, tailoring information to individual needs, and by providing patients with tools to engage in their own health care management. For care teams, digital technology can add value through early detection of postoperative complications, proactive surveillance of health data for postoperative patients and patients on waiting lists, higher compliance with rehabilitation programs, and reduced length of stay. Digital technology has the potential to improve patient satisfaction and outcomes, as well as potentially reduce hospital length of stay and the burden of disease associated with postoperative morbidity.

## Introduction

Health care delivery is changing from paternalistic models toward more participative and patient-centered models in which patients, as consumers of health care services, are viewed as active rather than passive participants in their own health care [1,2]. The growth in patient-centered care delivery combined with the rising costs of health care have perhaps not unsurprisingly been matched by a proliferation of patient-centered technology [3-8]. In this research, we explore how care teams can utilize digital technology to support patient-centered care for better outcomes and higher patient satisfaction in total knee replacement (TKR).

TKR is an increasingly popular elective surgery for people wanting relief from knee pain and loss of lifestyle because of reduced functionality. Osteoarthritis is the primary diagnosis of knee replacement patients (accounting for 97% of surgeries) [9]. The average age of patients undergoing TKR in Australia is 69 years [10]. When recommended, TKR is generally effective in reducing knee pain and improving functionality [11,12]; however, despite many successes, there is still a relatively high degree of patient dissatisfaction following TKR. A 2007 survey of 10,000 TKR patients in the United Kingdom showed that only 82% of TKR patients were satisfied with their outcome 12 months after surgery [13]. More recently, a 2012 systematic review of journal articles on postoperative outcomes for TKR indicated that as many as 10% to 34% of TKR patients continue to experience long-term pain after knee replacement [14].

In this paper, we explore the facilitation of patient-centered care using digital technology in a clinical setting for the delivery of TKR. Specifically, we draw on the concept of cocreation of value based on service dominant logic [15-17]. Service dominant logic is a marketing perspective that calls attention to the importance of services in creating value for consumers whereby value, or the utility of a service, is determined by the customer [16]. Although TKR, akin to other health services, does involve the exchange of goods (implant, medication, and consumables), it is predominantly a service provided by hospitals, clinics, and care teams [2,18-20]. Similar to other services, health care requires the active participation of consumers (ie, patients) [18]. The value of the service for the consumer is created, or rather cocreated, with the service care team. Value or utility of a service to customers is uniquely determined by the customer [15]. TKR surgery is a means to an end. The value or utility of the surgery is determined by each individual, and the value for the patient could be about playing or being able to play with grandchildren, going on an overseas trip, or being able to continue to play golf. Interactions between service care teams and consumers are opportunities to add value. The way in which interactions are managed can facilitate (or destroy) consumer value [21-24]. Digital technology has the potential to enhance the delivery of health care services by adding value to the interactions and communications between patients and their care teams [25-28].

In this paper, we explore the TKR journey from start (first referral to a surgeon) to finish (12 months after surgery) to understand the disparate needs of stakeholders and identify opportunities for digital technology to add value to TKR health care service delivery by enhancing the cocreation of value.

## Methods

### Design: Qualitative Study

Ethics approval was obtained from the *Commonwealth Scientific and Industrial Research Organisation* (CSIRO) Health and Medical Human Research Ethics Committee: low risk review panel. Qualitative data were collected using focus groups and interviews conducted in Queensland and New South Wales, Australia from June 2015 to September 2015. In total, 34 people (patients, general practitioners [GPs], and clinicians) participated in the research. Participants were recruited based on a maximum variation sampling approach [29] to ensure a diversity of opinion and experience. All focus groups, interviews and discussions were audio-recorded and transcribed. Qualitative analysis was carried out using NVivo software (QSR International) to assist in the management of the coding task. Thematic analysis was used to identify emerging themes [30].

#### Study Population

Participants (N=34) included three different target groups: patients (n=11), GPs (n=8), and clinicians (n=15), defined in this research as surgeons (n=10), research fellows (n=2), and physiotherapists (n=3).

Patients and GPs were invited to attend structured focus groups and clinicians were invited for interviews or discussions. In the following sections, we detail the participant recruitment process and how data were collected. For each group of participants, we outline the key questions. A more comprehensive list of focus groups and interview questions is available in Multimedia Appendix 1.

### Methods of Data Collection

#### Focus Groups

Focus groups (n=17) were used to collect data from GPs and patients. For convenience and expediency, we contracted two different Sydney-based medical market research companies to recruit participants for general practitioner (GP; n=8) and patient focus groups (n=9) because market research companies have access to a large number of GPs and patients with the relevant history of TKR needed to convene a focus group. The focus groups were conducted by CSIRO researchers and lasted around 1 hour long. All participants signed consent forms.

**Table 1:** General practitioner details.

Demographics		Bulk billing (Medicare), n	Private, n
**Gender**			
	Male	1	3
	Female	1	3
**Age (years)**			
	35-44	1	2
	45-54	1	2
	55+		2
**Years’ experience**			
	6-10		1
	11-19	2	2
	20+		3
**Hours worked**			
	21-40	1	1
	41-60		6

GP focus groups (n=8): GPs were screened based on gender, age, years in general practice, weekly patient care hours, proportion of public patients (100% funded by Medicare), and frequency of referral of patients for TKR (see Table 1). The aim of the GP focus group was to understand more about the role of GPs in TKR in Australia because patients cannot consult a surgeon without a referral from a GP. Questions included recounting some of their experiences in referring patients for TKR, with examples of different types of patients, patient outcomes, and differences in referral and treatment between private and public patients. GPs were also asked about any treatments they recommended presurgery, such as exercise or weight loss, as well as their role in postsurgery.

Patient focus groups (n=9): patients were screened based on gender, age, type of knee surgery, principal diagnosis, year of surgery, patient type: private or public, and choice of rehabilitation. Table 2 summarizes patient demographics. A total of 10 patients were recruited, but one did not attend. The aim of the patient focus group was to understand the experience of TKR patients as a basis for developing an app or technology solution to assist them through TKR. Patients were asked about how they came to consider and make the decision to undergo TKR surgery, specifically asking about the process they went through and what sort of information they needed along the way to make decisions or to fulfil their role as a patient. Patients were then asked to describe their experience of TKR surgery, including the hospital stay, the rehabilitation, and recovery from surgery. Patients were also asked about what might have been done differently or better. Because the experience of TKR was uniformly positive among the 9 patients in the focus group, researchers used convenience sampling (word of mouth) to recruit and interview a further 2 patient participants to capture a more balanced view of the patient experience.

#### Interviews or Discussions

A total of 17 people, including patients (n=2) and clinicians (n=15), participated in semistructured interviews or discussions. Discussions refer to interviews involving more than one participant. Participants were recruited using convenience sampling, people known to either the research team or the research sponsor. The research included both public and private sector practitioners. Participation in interviews or discussions with surgeons and physiotherapists was opportunistic and self-organizing. Participant numbers varied between 1 and 8 attending depending on availability and opportunity on the day. One interview was conducted by phone, all others were face-to-face. The duration of interviews varied from 25 to 120 min dependent on the size of the group.

Patients (n=2): for maximum sample variation, it was important to explore bad patient experience or service failure. Researchers used convenience sampling (word of mouth) to recruit 2 patient participants who were interviewed together. Both had postoperative complications, multiple revisions and knee replacements. The interview covered some of the same ground as the focus group questions but focused on the experience of, and reasons for, service failure.

Clinicians (n=15): clinician interviews or discussions included orthopedic surgeons (OS, n=10), research fellows (n=2), and physiotherapists (n=3). Participants were recruited from both Queensland and New South Wales, see Table 3. Although interview’s and discussions followed a broad direction, they were less structured than the focus group because of the multidisciplinary nature of groups, the number of attendees, differences in patient base (private and public, local or remote), as well as interstate differences in hospital practice. The aim of the interviews with clinicians was to understand the process of TKR. Clinicians were asked about each step in the TKR timeline (for both private and public patients) from referral to 12 months post surgery, which is when TKR patients are deemed to have fully recovered from surgery. Particular emphasis was placed on understanding the communication and information exchange between clinicians and patients. Clinicians were asked to explain how and when information was provided to patients and discuss issues where patients did not fully understand or follow instructions. Clinicians were also asked to describe types of patients, as well as their experience of patient compliance. Finally, surgeons were asked to discuss how they managed patient satisfaction and to describe circumstances leading to poor outcomes.

**Table 2:** Patient demographics, including both focus group (n=9) and interview participants (n=2).

Demographics		Patient types		
		Private patients, n	Medicare patients, n	Covered by workers compensation, n
**Location**				
	Queensland	1		1
	New South Wales	7	1	1
**Gender**				
	Male	4		1
	Female	4	1	1
**Rehabilitation**				
	Inpatient rehabilitation hospitals	5		1
	Outpatient rehabilitation	1	1	
	In-home physiotherapy			1
	Local physiotherapy clinic	1		
	No formal physiotherapy	1		
**Age (years)**				
	50-59	4		1
	60-69	3	1	1
	70-79	1		

**Table 3:** Clinicians’ breakdown.

Demographics		Clinicians		
		Surgeons, n	Fellows, n	Physiotherapist, n
**Location**				
	Queensland	7	1	2
	New South Wales	3	1	1
**Gender**				
	Male	8	1	2
	Female	2	1	1
**Health care regime**				
	Public	2		1
	Private		1	2
	Public and private	8	1	

## Results

Two themes emerged from patient and clinician data on the experience of TKR. First, for patients TKR is not an event but a long journey (12+ months). The journey is clearly marked by stages with different interactions and opportunities for cocreation at each stage. Second, effective communication and information flow between patients and clinicians is a key cocreation task in TKR, irrespective of the stage of the journey.

### Theme 1—The Patient Total Knee Replacement Journey

It was clear from the way patients and clinicians described TKR that it is not an event but a journey of 12 months or more, which officially starts when a patient obtains a referral to an orthopedic surgeon (OS) from their local GP. Seven stages of cocreation were identified: referral, consultation, prehabilitation, perioperative, hospital stay, rehabilitation, and postoperative (Table 4).

#### 1. Referral

In Australia, patients require a referral from a GP to access an OS. The GP referral marks the start of the patient’s TKR journey. Most patients will have seen their GPs over the years for treatment and management of the increasing knee pain and loss of functionality typical associated with osteoarthritis before getting a referral for surgery. Some patients anxious to avoid loss of lifestyle request TKR early, others, because of fear of surgery or concerns about the outcomes, leave it too long:

Some want it way too early. They just think, oh, a little bit of a twinge, I might get a new knee.

GP 2

I had another guy the opposite way who put his knee replacement off for ages and ages.

GP 8

The referral stage is marked by information seeking and decision making. Patients must decide if they wish to pursue surgery, and private patients can also decide which surgeon they want to be referred to. To support decision making, patients are very active in information seeking, principally using the Internet, GPs recommendations, and increasingly via their social networks. Patients research the medical procedures involved, the experience of TKR, and prospective surgeons. GPs reported that private patients are increasingly assertive in exercising their rights as consumers of health care services. They are actively requesting TKR surgery to avoid loss of lifestyle and/or referral to a specific surgeon or a number of surgeons based on their research:

Lots of people I know they’ve had knee replacements; they were all very successful, so I just took their word, and their recommendations.

Patient 8

**Table 4:** The stages of the patient journey.

Stage	Cocreation network	Patients role	Timelines and details^a^
(1) Referral	GP^b^, social network^c^	Information seeking. Decision making: decision to consider surgery.	Private: 2-12 weeks; Public: 12 months.
(2) Consultation	Administrative staff, OS^d^	Understanding information, deciding on surgeon (private patients), risk management, establishment of a relationship of trust with surgeon.	Private: 2-4 weeks; Public: approximately 12 months.
(3) Prehabilitation	Physiotherapist	Being fit for surgery and exercise completion.	Private: more likely to go straight to surgery; Public: emphasis on home exercise therapy.
(4) Perioperative	Administrative staff, occupational therapist, physiotherapist, registered nurse (RN)	Preparation for hospital admission and discharge.	Public and private: 2 to 4 weeks before surgery.
(5) Hospital stay	Administrative staff, OS, RN, physiotherapist, occupational therapist, social network	Following instructions and providing feedback on progress.	Public and private: walking day 1, discharge day 3 to 5.
(6) Rehabilitation	Physiotherapist	Exercise completion and providing feedback on progress.	Private: in- and outpatient rehabilitation and private physiotherapy; Public: outpatient rehabilitation, limited public funding for private physiotherapy.
(7) Postoperative	Administrative staff, occupational therapist, RN, GP	Detecting and reporting complications. Following medication. Staying positive.	Private: scheduled appointments, removal of surgical clips at clinic or by OS at 2 weeks, OS at 6 and 12 weeks and at 12 months; Public: fixed schedule, removal of surgical clips at clinic or GP at 2 weeks, OS at 6 and 12 weeks and at 12 months.
(8) Complications (restart from perioperative)	GP and/or OS		Private: more access to OS over and above scheduled appointments; Public: more gatekeepers.

^a^There is some variation in practice especially in private surgeons, the time lines suggested reflect common practice.

^b^GP: general practitioner.

^c^family and friends.

^d^OS: orthopedic surgeon.

Public patients have less, if any, choice in their surgeon and are therefore more passive consumers at this stage of their TKR journey. The time between referral by a GP and the first consultation with a surgeon varies based on health cover. Private patients will have their first consultation with an orthopedic surgeon usually between 2 to 4 weeks after referral. However, because TKR is an elective surgery, public patients can wait up to a year to see a surgeon. Waiting lists are problematic because GPs try and get public patients onto to a waiting list early so that by the time they need surgery, they will already be on a surgeon’s list. This, however, creates additional work for surgeons who must review all patients on their lists yearly. However, these annual reviews are often the only thing assuring patients that they have maintained their position on the waiting list.

#### 2. Consultation

The consultation stage for patients is about risk assessment and forging a relationship of trust with their orthopedic surgeon. Importantly, during this stage, patients establish expectations about the outcomes of TKR, which in turn affects their subsequent satisfaction with the surgery:

[S]atisfaction is directly linked with expectations. So if there are those conversations preoperatively about this will take you 12 months or 2 years to get over, I think they are much more willing to accept that that’s the case, rather than being dissatisfied for the first 12 months.

Fellow 1

In these initial consultations, establishing a relationship with the surgeon is very important. Patients must be comfortable with and have faith in the surgeon’s ability to deliver the outcomes they expect:

I went to a surgeon first, someone who had done my father’s knee...he wasn’t the surgeon I ended up with because I couldn’t stand the guy. I wasn’t going to let him cut me open.

Patient 1

#### 3. Prehabilitation

Once a decision to undergo surgery is taken, private patients have a 2 to 8 week wait for their surgery to be scheduled. Private surgeons tend not to recommend prehabilitation:

Very rarely would someone of mine go in for prehab[ilitation]...I don’t know if I’m right or wrong—but I tend to reserve the rehab[ilitation] for postsurgery.

Surgeon 4

In the public sector, waiting time can be over 12 months. GPs, physiotherapists, surgeons, and public services use this time to promote prehabilitation:

If they come in at a reasonable period before their surgery date, for instance, if they’ve got, say, 4 to 6 weeks beforehand, it gives us the opportunity to possibly improve, say, their knee extension prior to surgery, because we know that will dramatically improve their walking ability postop.

Physiotherapist 1

I don’t think you will come to a knee replacement for some time and what I’m going to do is plan to see you in a year but I want you to do [the exercises] and I’m going to write to the GP and say if there’s any major changes or a problem I’m happy to see you earlier. So they’ve got a prehabilitation process.

Surgeon 10

#### 4. Perioperative

Once surgery is scheduled, the perioperative stage begins. This stage is information rich. Hospitals and care teams need to communicate with and collect information from the patient with a view to facilitating a cost-efficient hospital experience and a timely admission and discharge.

#### 5. Hospital Stay

Patients admitted to hospital have their surgery and are typically discharged 3 and 5 days after surgery. Private patients in large metropolitan areas have the option of being discharged to inpatient rehabilitation clinics for a 1 or 2 week stay where inpatient physiotherapy is delivered. Public patients who have less access to rehabilitation services post surgery may be kept in the hospital longer if the surgeon feels they might benefit from a few days extra of inpatient physiotherapy:

The big difference between public and private, as far as how long they stay in hospital, is really dictated by access to rehab. Private patients can get into rehab, whereas public patients...rehab is almost impossible to find...at least they’re receiving some inpatient physio...before going home.

Surgeon 2

#### 6. Rehabilitation

Rehabilitation begins in the hospital and continues into the postoperative stage. In-patient physiotherapy starts on the day after surgery when patients are expected to start walking with aids. In addition to walking, patients also have to complete exercises designed to improve knee extension (straightening), range of movement (bending), and function (walking, steps, and running). Rehabilitation continues after discharge at inpatient or outpatient clinics, or with local physiotherapy services. Proximity to rehabilitation facilities or physiotherapists and private health care cover determine access to rehabilitation. However, all physiotherapists recommend in-home exercise and self-management of prescribed in-home exercise as an important part of rehabilitation.

#### 7. Postoperative

Patients will have their wound assessed and clips removed 2 weeks after surgery by their GP in a clinic or by their surgeon. They also typically see their surgeon at 6 weeks to check for wound infection and at 12 months to assess recovery and the performance of the prosthetic. In the postoperative stage, the patient is responsible for detecting and reporting postsurgical complications, including wound infection, clots, and deep vein thrombosis. Although the risks of complications post surgery are low, the consequences are very high, which can cause some uncertainty and anxiety in patients:

Well, with things like that, a lot of patients at 6 weeks are worried, “My knee is hot and red.” And [knees post surgery] are all hot and red.

Surgeon 1

### Theme 2—Information and Communication Flow

Two-way communication between patients and care teams is essential in the TKR journey. Clinicians, including surgeons, physiotherapists, and others, need to communicate information effectively to manage patient expectations. Communication is also essential to ensure informed consent and decision making and to ensure patients meet the surgeon’s and hospital’s expectations for self-management pre and post surgery. Equally, patients need to communicate information effectively to care teams for accurate diagnosis and treatment in the consultation and postoperative phases. Our analysis revealed issues with information flow, gaps in communication, and communication problems.

#### Information Flow

Care teams and hospitals have to communicate the same information to patients repeatedly. This requires efficiency in communication. Due to the limited amount of face-to-face time, printed information is the principle means of supporting information communication to patients at all stages of their TKR journey:

When every patient books for an operation, we give them [an information pack] this is a preloaded what paperwork is required, the consent form, the hospital admission paperwork, thing about knee replacement et cetera, et cetera.

Surgeon 5

I got a booklet…and [the surgeon] explained a little bit also with an instrument of some sort; I don’t know. Showed how the joints fit together, and he gave me the brochure and sent me off.

Patient 4

Increasingly hospitals are organizing presurgery education or assessment sessions during the perioperative phase. These sessions often combine group information sessions and one-on-one consultations with nurses, physiotherapists, and occupational therapists:

Well, I have worked in two private hospitals, and they had significant differences in their length of stay. The big difference has been the patients who have been preoped have everything organised, arranged, and mostly their expectation of discharge. So they come in and they go, I’m having a total knee replacement, I’m going to be in, my surgery is on Monday, I’m going to be home Friday.

Physiotherapist 3

However, interview data revealed that patients do not necessarily absorb, retain, or act on the information provided:

We always inform the patients not to take antibiotics. But I do the review at 2 weeks; a lot of people are full of antibiotics for unnecessary reasons.

Surgeon 1

There was one patient that will forever stick in my mind. She was in absolute tears when I called her, and she was distraught...I found out that she...thought that she had to stay in the house. [S]he thought that she was trapped in the house until she went back to see the GP at 2 weeks and the surgeon at 6 weeks.

Physiotherapist 2

Information can also allay fears and concerns:

We often have to ask them, have you done the [presurgery] education? And you know the ones that haven’t, because...they’re apprehensive about getting out of bed.

Physiotherapist 2

Physiotherapists also provide patients with a lot of information. Physiotherapist’s explain to patients how to modify everyday activities to cope with limited functionality (eg, how to climb stairs safely and the best way to get in and out of bed) and prescribe exercise plans which include the type of exercise, the number of repetitions, and the recommended frequency. Importantly, they also teach patients to perform exercises correctly. In addition to providing information, physiotherapists also have to motivate the patient to complete their exercise program:

So a lot of them get given information sheets and stuff like that, they watch videos in there as well and then get given a whole lot of stuff to do at home, so I think a good rehab is a rehab that will encourage them to be self-motivated rather than just doing 4 hours of rehab a week while at rehab.

Physiotherapist 1

Patients must also convey information to care teams. Although patients are not medical experts, they are the only ones who can communicate what they feel and experience, therefore giving them *expert* knowledge. Surgeons and physiotherapists need quality feedback from patients for their diagnosis and ongoing care. Pain and sleep are two important variables that help surgeons and physiotherapists assess progress. Sleep quality is directly associated with pain, and care teams use both to understand patient progress. Other key information patients are required to provide to the doctor is physical functionality:

I think it’s critical…to take a history about physical status, what are they doing and I [cover] hills and stairs, crouches and squats, kneeling and ladders, running and jumping,…the way you answer me will tell me where in the knee the problem lies…each of those activities, and the way you weight them helps me to know where the problem is.

Surgeon 10

The decision to operate is—it’s their pain level, it’s their function, it’s the, can you walk—I can’t walk around the shops, I can’t sleep…and my usual statement to patients is, “You have your operation when you are ready.”

Surgeon 1

#### Cocreation Styles

In addition to the cocreation tasks associated with information gathering and decision making, patient’s personalities and life experience mean that they establish different types of relationships with their surgeons and physiotherapists. The type of relationship they establish is particularly apparent in the postoperative stage, where lack of expertise or experience and the high cost of undetected complications makes patients anxious and fearful:

Rare complications when you have them, they’re disastrous (64.10) infected prosthesis, it’s terrible. I’ve had patients who’ve had a protracted postoperative of 6 months said, I wish I never set my eyes on that surgeon.

GP 3

Because if you wash [infected joints] out in the first few weeks, you may be able to save the joint. So it actually matters a lot. And the main reason, probably we see the thousands [of patients at] 6 weeks is to pick up the one [infection].

Surgeon 1

Public and private patients generally see their surgeons at 6 weeks and at 12 weeks. In the postoperative stage, private patients have more access to their surgeons. They are more readily able, and perhaps feel more entitled, to communicate directly with their surgeon:

There’s usually a heightened sense of awareness and patients are paranoid about clots and infection, so most people will ring at the drop of a hat if they’re worried...if someone’s worried I’d rather see them.

Surgeon 9

Public patients are generally encouraged to see their GP if they have concerns about postsurgical complications, despite a number of surgeons reporting that GPs lacked knowledge to correctly treat postsurgical infections. Over and above differences in health care cover, differences in personality and communications style result in both under- and overreporting.

Overreporting occurs when patients are overanxious and frequently contact the surgeons directly or via the practice receptionist for private patients or via the GP for public patients. Clinician time is limited, so to relieve pressure on care teams and GPs, practice receptionists can act as gatekeepers, filtering requests. On rare occasions, their lack of clinical expertise, coupled with patient’s limited ability to communicate their condition, could result in genuine complications being overlooked:

When I go in there the message that [my surgeon] had [from his receptionist] was that I had nicked the top of the scar. It was wide open, I had to have a tourniquet to stop the bleeding...that was the arrogance of a front line staff member who had not even passed the message on.

Patient 11

Underreporting also occurs and is equally problematic. Patients who do not want to bother the surgeon will wait until the next appointment to raise concerns that again can exacerbate outcomes:

What I say to people is...“See you in 6 weeks.” And then they sit there for 2 weeks with pus pouring out of their knee, because they are seeing the doctor at 6 weeks...so whatever happens in between, they just hold on to it.

Surgeon 1

Most patients tend to follow medical advice. Many patients referred to themselves as a “good patient,” following care team advice and instructions, in particular with respect to medication and exercise:

I think I was an excellent patient...so I did all the things I was told to do [in rehab] because I just wanted the best recovery I could have and I just thought their exercises, their knee clinic I think they called it, it was just really good.

Patient 3

Reasons for not completing rehabilitation or home exercise included access to rehabilitation, having to rely on others to drive them to a clinic, the need to return to work, and pain:

The rehab, getting that bend in the knee, I can still remember tears streaming down my face trying to do what they were telling me to do, to get that particular degree that they wanted.

Patient 3

I was really concentrating on exercising and that, while I was in the hospital…then I wanted to get back to work, because I was totally bored. So I didn’t do as much rehabilitation as what I should be doing.

Patient 7

Due to pain and difficulty of completing exercises, some patients (Patient 11) developed very negative attitudes toward their physiotherapists describing them as “overzealous, rude, and condescending.”

## Discussion

### Principal Findings

This research explored TKR patient-clinician interactions looking for opportunities for digital technology to enhance cocreation and add value at different stages of the TKR journey. Technology can add value by enhancing and increasing opportunities for two-way communication between clinicians and their patients.

#### Communication From Clinician to Patient

Results showed that interactions between clinicians and patients are time-poor but information-rich, and patients do not necessarily retain or recall the information or instructions given to them by their care team because information given by clinicians, and in particular surgeons, to their patients is often technical and complex [31]. About half a surgeon’s consultation time is spent explaining the medical condition, treatment options, and surgical procedure; a good deal of which is required to meet legal obligations [31]. Although this information is necessary for patients to understand their condition, the risks associated with treatment options, and to provide informed consent to treatment plans [31-34], it has been demonstrated that patients have very poor recall of information provided to them by clinicians, especially if, as with TKR, they are older, in pain, or anxious [35]. Patients also have selective information retention and generally have higher recall of information about the diagnosis than about the treatment options [35]. Similarly, with rehabilitation, patient recall of exercises, sets, and repetitions is particularly problematic for older adults, and recall is not substantially improved by the provision of a memory sheet [36]. Results showed a clear role for digital technology to add value through improved communication and information flow between clinicians and patients. Digital technology can facilitate the following:

- *Deliver the right information at the right time*. Mobile phone features such as short message service, push notifications, reminders, tasks, and alarms are well suited to supporting real-time information delivery and “just-in-time” access [4,8]. The results clearly identified different information needs at different stages of TKR. Technology-supported information delivery can deliver the right information at the right time, making it both easier to absorb and readily accessible to add value through improved patient recall and compliance. Reminders have been successful in increasing adherence for routine daily tasks such as medication management [4]. Mobile notifications may also increase adherence to home-based physiotherapy, as research has shown that setting regular times for exercise and integrating exercise into routine is more likely to encourage successful completion [37]. Interactive tasks or checklists could potentially help patients prepare for hospital admission, complete rehabilitation plans, and take medication as prescribed.
- *Convey complex information in a more engaging way, including text, imagery, audio, or video* [36], thereby adding value through better patient understanding of treatment and rehabilitation. Mobile phone apps with videos could support demonstrating the surgery and the treatment options, and video is a great medium for providing exercise demonstrations to support safe and correct exercise at home [38-42].
- *Enhance motivation and compliance with exercise programs through interactive gaming and rewards systems.* Gamification is a commonly used engagement strategy to increase motivation by providing positive feedback to users, which in TKR could include compliance with physiotherapy and achievement of functionality milestones. Achieving functionality milestones could support patients’ sense of positive progress toward recovery.
- *Increase the number of interactions (albeit indirect) and thereby the opportunities for adding value between care team and patients*. Encounters and interactions with clinicians are dictated by standardized care pathways that determine duration and number of appointments. Push notifications and just-in-time delivery of information can add value by indirectly increasing the communication or interactions from care team to patients between appointments. Mobile phone apps can also provide patients who cannot or do not have access to rehabilitation services with support in completing their rehabilitation. A mobile phone app for cardiac rehabilitation has been proven to increase uptake, adherence, and completion of patient rehabilitation for patients who have had a heart attack in comparison to face-to-face outpatient clinic [39,40].
- *Tailor information to individual needs to deliver personalized solutions.* Mobile technology can ensure that individual patients receive personally relevant information and/or can make choices in how they receive the information and which support tools they use. Mobile phone apps can also potentially match different behavioral interventions to different cocreation styles to optimize outcomes [24].

#### Communication From Patient to Clinician

Patients have an *expert* role in that only they can describe what they are feeling and experiencing. This patient input is a crucial element of clinical diagnosis and treatment [32,43,44]. Results show that technology could add value to patient clinician communication by supporting patients in their role of an *expert* to better and more accurately communicate how they are feeling and what they are experiencing. Self-reported information can be difficult for patients to recall, inconsistent over time, and difficult to convey [45]. Research shows that patient self-reports on sleep and pain are unreliable. For participants with fair or poor health, no correlation was observed between subjective and objective sleep measures [46], and patients with a high social desirability bias (desire to be viewed favorably by their surgeon) report higher level of pain [47]. For patient clinician communication, digital technology can facilitate the following:

*Enhance the quality and frequency of patient communication with their care team using self-monitoring tools and wearables.* Self-monitoring using mobile phones could assist patients in recalling the timing of events and improve accuracy and validity of self-reports on pain or recovery progress with text-based diaries, photo or video blogs, tools to track pain, medication use, mood, physiotherapy, knee range of motion, and more. This type of reporting could be further enhanced with the use of wearable technologies such as activity trackers, body worn sensors, or Bluetooth-enabled thermometers or scales. By linking data from digital technology to a Web portal accessible by the care team, daily progress data could be used to flag patients not progressing as expected, allowing clinicians to move appointments forward as needed. Remote monitoring of progress could potentially increase motivation and compliance as patients extend the role of *good patient* to their self-management tasks, knowing that they are been observed. This approach could also be used for waiting list management to supplement the yearly orthopedic consult.

*Assist in the early detection of postoperative complications and reduce patient anxiety*. Results showed that the most critical time for effective patient communication is the postoperative period (0-12 weeks post surgery) because even though the risk of infection and complication after TKR are low, the resulting morbidity is high, and patients are naturally anxious. With only limited access to surgeons (3 postoperative visits in 12 weeks), it is largely the patient’s responsibility to detect and report postoperative complications and infections. Results showed that patients both under- and over-report postsurgical complications. There is very clearly a role for technology to add value in the detection of postsurgical complications to help reduce patient anxiety and uncertainty and improve patient outcomes. Solutions might include telehealth services for the 12-week postoperative period or intelligent technology such as smart wound dressings, wearable devices, and heat detectors for infections [48,49].

### Strengths and Limitations

The strength of this research is that it provides a multistakeholder perspective on how care teams can use digital technology to add value to patient clinician interactions and in so doing, potentially improve patient experience and satisfaction. Limitations of this research include the limited generalizability of qualitative data. We also acknowledge an overrepresentation of the private sector perspective (patients and care teams), even though 70% of TKR in Australia occurs in private hospitals [50]. Although research was conducted in two major cities, Sydney and Brisbane, this study is not necessarily representative of TKR journey in whole of Australia, as only 60% of TKR surgeries occur in capital cities [51]. Focus groups conducted in remote and less affluent areas may have yielded different patient experiences because access to TKR and physiotherapy are affected by proximity to hospitals and rehabilitation facilities, as well as affordability. Finally, this research was cofunded by a commercial partner who has engaged CSIRO to conduct this research independently on their behalf. CSIRO was solely responsible for decisions on the study design, analysis, and interpretation of data. This research has led to the design and development of a digital orthopedic rehabilitation platform, which is being evaluated through a multihospital randomized controlled trial and registered with the Australian New Zealand Clinical Trials Registry (ACTRN12616000504415) [52].

### Conclusions

Digital technology has the potential to enhance the current model of care for TKR, adding value for all stakeholders through increased and improved communication and information flow. For patients, digital technology could enhance information retention and recall, support the patient as an *expert*, reduce anxiety in the postoperative stage, improve recovery through improved adherence to rehabilitation, and increase satisfaction through supporting personal agency and perceived control. For clinicians, digital technology can enhance the communication and information flow between care teams and patients to improve patient compliance, outcomes, and satisfaction. For health care providers, digital technology can assist in managing waiting lists, reduce length of stay because patients are better prepared for admission and discharge, and reduce morbidity and burden of disease through early detection of postoperative complications. Digital technology could also potentially reduce the cost of service delivery [53] without compromising patients’ outcomes. Although *patient-centered* care is by definition not *technology-centered* care [54], technology can nevertheless assist in the delivery, reinforcement, and accessibility of information if the focus of digital technology design and development (eg, apps) is value-driven and patient-centric [25].

## Appendix

Multimedia Appendix 1 Interview questions.

## Abbreviations

GP: general practitioner
OS: orthopedic surgeon
TKR: total knee replacement
CSIRO: Commonwealth Scientific and Industrial Research Organisation
