# Supplementary material for: Total Knee Replacement and the Effect of Technology on Cocreation for Improved Outcomes and Delivery: Qualitative Multi-Stakeholder Study
Source: J Med Internet Res. 2018 Mar 20;20(3):e95. doi: 10.2196/jmir.7541 (PMC5883076; doi:10.2196/jmir.7541)
Supplement: Multimedia Appendix 1 [file jmir_v20i3e95_app1.pdf]

APPENDIX 1

GP - INTERVIEW GUIDE

Referral of patients to orthopedic surgeons

- What are the GPs' pre-surgery responsibilities to total knee replacement patients?
- When you refer a patient to an orthopedic surgeon, what is the referral based on?
- What is a non-surgical intervention for knee pain before referring someone to an orthopedic specialist?
- In making assessments about suitability for TKR how reliable are patients self – reports?
- Does the treatment or referral process change when for people without private health cover or Workcover?
- How do patient care teams (surgeon, GPGP and physiotherapist) communicate?
    - Medication

Post-surgery

- What happens post TKR? When do GPs post-surgery responsibilities to Knee Replacement Surgery patients start and what does that involve?
- How do GPs assess progress?
- When a patient returns to your care post-surgery what are your goals with them?
- If you could get objective information from the patient what would be of most interest to you in assisting patient recovery?

Measuring success

- What constitutes successful patient outcomes?
- What are the implications of clients not engaging in rehabilitation? (physical, emotional, economic)

SURGEON - INTERVIEW GUIDE

General

- Can you tell us a about the types of patient that are potential candidates for knee replacement surgery?
- Gender, age range (% age split), expectations

Decision making

- When patients are referred is it with total knee replacement in mind or is it less specific?
- What factors do you consider before recommending total knee replacement surgery?
  - Is it collaborative decision?
  - Is the process lengthy?
    - How do you manage patient expectations?
  - How accurate a patient self-reports (exercise, mobility, pain history, etc.)
    - Are patients open to non-surgical alternatives?
    - How common is it for you to be asked to provide a second opinion for someone considering knee replacement surgery? Why are they seeking a second opinion?
      - Do the treatment options differ for people with/without private health cover or Workcover?

Pre-surgery

- What concerns do patients have pre-surgery? How do you deal with these?
- Do patients come to you well informed about knee surgery?
- How useful is information on the Internet to patients?

Recovery

- How do you monitor patients post-operatively?
- What information do you require from patients to assess progress?
- How do patient care teams (surgeon, GP and physiotherapist) communicate?
  - What are the barriers to successful surgical outcomes?
  - What are major causes of patient dissatisfaction?
  - What are the most common complications from TKR?
    - What about mobility? How do your currently assess mobility?

PATIENT FOCUS GROUP INTERVIEW GUIDE

Decision making

- How was it you came to consider total knee replacement?
- Did your GP, physio or surgeon suggest non-surgical interventions?
- What or who influenced your decision making?
- Did you do any Internet searching prior to surgery?
- From whom did you get support/help over this period?
- What were your expectations and hopes for recovery?
- If you hesitated to commit to the surgery, for what reasons did you hesitate?

Post surgery

- What was most difficult part of the recovery?
- What type information did you receive from your physiotherapist to assist you with your rehab (Pamphlets, access to website, recommendations for websites/apps)?
- What kind of difficulties did you encounter in completing rehab?
- What were the high points (achievement) of your recovery?
- From whom did you get support/help over this period?
- Are you happy with the outcome?
- What would you do differently if you could do it again?

PHYSIO – INTERVIEW GUIDE

Prehabilitation

- Do you get referrals for prehabilitation?

Rehabilitation

- How do patients access rehabilitation post-surgery? Private/public
- What is your experience is completion rate of rehab?
- What are some of the major barriers to successfully completing rehab?
- Do patients complete rehabilitation?
- What do patients report as reasons for not completing rehab?
- How reliable are patient self-reports?
- What are the big motivational enablers for patients?
- Do you prescribe a treatment protocol for patients?
    - How does it vary between patients?
- How do you assess progress?
- How do you assess mobility and functionality?
